# Treatment patterns, adherence to international guidelines, and financial mechanisms of the market access of advanced breast cancer therapy in Bulgaria

**DOI:** 10.3389/fpubh.2023.1073733

**Published:** 2023-03-03

**Authors:** Stephanie Karanyotova, Branimira Topova, Elina Petrova, Peter Doychev, Eliana Kapitanska, Guenka Petrova, Zornitsa Mitkova, Maria Dimitrova

**Affiliations:** Department of “Organization and Economics of Pharmacy”, Faculty of Pharmacy, Medical University of Sofia, Sofia, Bulgaria

**Keywords:** advanced breast cancer, patient access, targeted therapy, availability index, compliance index

## Abstract

**Introduction:**

Breast cancer is the most common type of cancer affecting women in Europe. Advanced breast cancer (ABC) poses a significant therapeutic challenge, and therefore, timely access to treatment is crucial. The aim of the present study was to evaluate the treatment patterns and patients' access to new therapies for ABC in Bulgaria.

**Methods:**

We conducted a retrospective study in the period 2008–2021. Based on the European Medicines Agency (EMA) database, we analyzed a number of medicinal products with marketing authorization for ABC in the last 13 years. Time to market access was evaluated as the degree of availability, which is measured by the number of medicines that are available to patients (availability index, AI), and the average time elapsed between obtaining a marketing authorization and time to inclusion in the Positive Drug List. Data were analyzed through descriptive statistics *via* Microsoft Excel version 10.

**Results:**

The average time to access was 564 days for targeted therapy. The availability and compliance index for chemotherapy and hormonal therapy in advanced breast cancer was 1, while the average AI for targeted therapy was 0.67. Patient access to targeted oncology therapy of ABC is above average for Europe and takes 1–2 years.

**Conclusion:**

Faster access is more evident for biosimilars. National regulatory requirements for pricing and reimbursement have a major impact on market access.

## 1. Introduction

Breast cancer (BC) is the most common type of oncology disease among women in Europe, and according to a report by the European Commission, breast cancer accounted for 355,457 new cases in 2020 (1). Breast cancer is the fifth leading cause of mortality. Survival rates of patients with breast cancer vary among different countries and mostly depend on the availability of established screening practices for early diagnosis and access to treatment (2). A comparison of the countries in the European Union (EU) reveals a lower breast cancer survival rate for countries in the Central and Eastern European region. In the Czech Republic, the breast cancer survival rate exceeds 80%, whereas it is 75% in Romania, 78% in Bulgaria, 74% in Estonia, and 77% in Lithuania (3). The 5-year breast cancer survival rates in Bulgaria increased from 70.9 to 78.3% between 2000–2004 and 2010–2014, but they remain lower than those found in the other EU countries (4). Diagnosis in the early stages increases the 5-year relative breast cancer survival rates-−99% for localized tumors and 86% for regional cancer, but, for advanced stages, the breast cancer survival rate falls below 30% (5). The established 5-year breast cancer survival rates in Italy are 100, 89.7, 71.4, and 29.1% for stages I, II, III, and IV, respectively (6). Epidemiological data show that 15–10% of the newly diagnosed patients are found to have a metastatic (advanced) disease and 20–30% of the early diagnosed patients are expected to have a metastatic relapse over time (7, 8).

Advanced breast cancer (ABC) is characterized by the presence of tumor cells in other organs such as the lungs, the liver, the brain, and the skin and poses a significant therapeutic challenge. The therapy aims to improve life expectancy and quality of life; therefore it is important that patients get timely access to treatment.

In 2005, the European Medicines Agency (EMA) imposed a centralized procedure for marketing authorization for all innovative oncology medicines and developed some additional procedures for early access in an attempt to provide timely access to oncology medicines. Further market access, however, also depends on the market size and the pricing and reimbursement procedures in the Member States which are currently not fully synchronized across the European Union and there remain local regulatory requirements. Recent studies show that there is significant variation in the time to market access to innovative medicines, especially in the oncology sector, and countries in which the decision for reimbursement is based on cost-effectiveness assessment and negotiations with national regulatory authorities have much longer time to market access (9–12).

Studies also showed that the average delay in time to market access and time to patient access for oncology medicines in Europe is approximately 400 days, but for many Eastern European countries, it is above the European average (7).

Reimbursement decision on new health technologies and new therapeutic indications in Bulgaria depends on several factors—analysis of the clinical efficacy, safety, and comparative effectiveness, cost-effectiveness, budget impact assessment, negotiations for price discount with the National Health Insurance Fund, and the reimbursement status of the same medicine in the European Union. The last mentioned factor requires an actual reimbursement of the new health technology in 5 out of 17 referent countries in the EU and at least one positive decision from the HTA bodies in the UK, Sweden, France, or Germany. This development prompted our interest to examine the current state and time to patient access to medicines for advanced breast cancer approved and reimbursed in Bulgaria.

## 2. Methods and materials

### 2.1. Design of the study

We conducted a retrospective analysis based on the EMA database (13) for medicinal products that received marketing authorization for ABC in the last 12 years (2008–2021). The indicators illustrating patients' access to therapy—*compliance with the international guidelines (guidelines compliance index, GCI) and availability index (AI)*—were calculated in the next stage. Finally, we reviewed the current requirements for pricing and reimbursement of oncology medicines based on an up-to-date regulatory analysis of the Ordinance on the terms, rules, and procedures for pricing and reimbursement of medicines (14) and Ordinance No. 10 for the terms, rules, and procedures for the reimbursement of medicinal products by the National Health Insurance Fund and the mechanisms and criteria for negotiations for price discount and budget predictability and sustainability (15).

### 2.2. Quantitative analyses

Patient access to these medicines in Bulgaria was evaluated through the following indicators:

*Compliance with the international guidelines (GCI)* is assessed as the number of medicines included in the national pharmaco-therapeutic guideline (16) and the number of medicines included in the guidelines of the National Comprehensive Cancer Network (NCCN) (17) and the European Society for Medical Oncology (ESMO) (18) based on the following formula:


$$\begin{array}{l} GCI=\frac{number\;of\;ABC\;medicines\;included\;in\;the\;national\;guideline}{number\;of\;ABC\;medicines\;included\;in\;NCCN\;and\;ESMO\;guidelines} \end{array}$$

*Degree of availability* is measured by the number of medicines available to patients (availability index, AI), and based on the following formula:


$$\begin{array}{l} AI=\frac{number\;of\;ABC\;medicines\;included\;in\;the\;Positive\;Drug\;List\;\left(PDL\right)}{number\;of\;ABC\;medicines\;with\;granted\;marketing\;authorization\;in\;the\;European\;Union\;\left(EU\right)} \end{array}$$

We also analyzed the average time elapsed between obtaining a marketing authorization and time to inclusion in the Positive Drug List in Bulgaria until December 2022 (19), measured in days for targeted therapy based on the methodology of EFPIA and IQVIA for “Waiting to access innovative therapy” (W.A.I.T.) concept (20).

Data were analyzed through descriptive statistics *via* Microsoft Excel version 10.

## 3. Results

During the observed period (2008–2021) in the European Union (EU), 44 medicines were granted marketing authorization for breast cancer indication, including metastatic breast cancer with predominantly targeted therapy.

One of the medicines (neratinib) currently authorized in the EU is suitable only for early-stage HER2-positive breast cancer. Four generic ibandronic acid-based medicines have indications for causing osteomodulator activity in breast neoplasm. Due to these circumstances, these INNs are excluded, and the current analysis is based on medicines with indications only for advanced and metastatic breast cancers (*n* = 39). Out of them, nine are generic medicines (23.08%), 13 biosimilar medicines (33.33%), and 17 innovative medicines (43.59%) for the treatment of advanced breast cancer.

Only nine medicines were prescribed for chemotherapy, of which eight were generic medicines, mainly docetaxel, capecitabine, and paclitaxel. Four of the trastuzumab and four of the bevacizumab biosimilars for targeted therapy are included in PDL.

A description of the type of therapy and the number and type of medicines for advanced (metastatic) breast cancer is provided in Table 1.

**Table 1:** The number and type of medicines granted with marketing authorization for 2008–2021 and their availability in the Bulgarian PDL for advanced breast cancer indication.

**INN**	**Number of medicines**	**Type of medicine**	**Included in PDL**
**Chemotherapy (n** = **9, 23.08%)**			
Paclitaxel	2	Generic + originator	√
Docetaxel	2	Generic	√
Eribulin	1	Originator	√
Capecitabine	4	Generic	√
**Hormone therapy (n** = **1, 2.56%)**			
Fulvestrant	1	Generic	√
**Targeted therapy (n** = **29, 74.36%)**			
**mTOR inhibitors**			
Everolimus	1	Originator	√
**HER2 inhibitors**			
Trastuzumab deruxtecan	1	Originator	√
Trastuzumab emtansine	1	Originator	√
Trastuzumab	6	Biosimilar	√
Pertuzumab	1	Originator	√
Pertuzumab/trastuzumab	1	Originator	√
**Tirosine kinase (TK) inhibitors**			
Lapatinib	1	Originator	√
Tucatinib	1	Originator	x
**VEGF inhibitors**			
Bevacizumab	7	Biosimilar	√
**CDK4/6 inhibitors**			
Ribociclib	1	Originator	√
Palbociclib	1	Originator	√
Abemaciclib	1	Originator	√
**PARP inhibitors**			
Talazoparib	1	Originator	√
Olaparib	1	Originator	√
**PDL-1 inhibitors**			
Atezolizumab	1	Originator	x
Pembrolizumab	1	Originator	√
**PI3K**α**-specific inhibitor**			
Alpelisib^*^	1	Originator	x
**Trop-2-directed antibody and topoisomerase inhibitor**			
Sacituzumab govitecan	1	Originator	x

^*^Alpelisib is not included in PDL but have has a registered price in Bulgaria.

The analysis of the degree of availability for the observed period shows that most of the medicines are available through the pricing and reimbursement (P&R) systems in Bulgaria. All generic medicines used for chemotherapy of advanced breast cancer are available in Bulgaria and are included in PDL.

In terms of compliance with international guidelines, the Bulgarian pharmaco-therapeutic guidelines for advanced breast cancer in Bulgaria follow the recommendations of the National Comprehensive Cancer Network (NCCN) and the European Society of Medical Oncology (ESMO). A summary of the degree of availability is presented in Table 2. Availability index (AI) is calculated according to INN and type of therapy for medicines for advanced (metastatic) breast cancer.

**Table 2:** Availability and compliance index in Bulgaria by INN and type of therapy for medicines for ABC in Bulgaria.

**INN**	**Availability index (AI)**	**Guidelines compliance index (GCI)**
**Chemotherapy**		
Paclitaxel	1	1
Docetaxel	1	1
Eribulin	1	1
Capecitabine	1	1
**Hormone therapy**		
Fulvestrant	1	1
**Targeted therapy**		
**mTOR inhibitors (mammalian target of rapamycin inhibitors)**		
Everolimus	1	1
**HER2 inhibitors (human epidermal growth factor receptor 2)**		
Trastuzumab deruxtecan	1	1
Trastuzumab emtansine	1	1
Trastuzumab biosimilar	1	1
Pertuzumab	1	1
Pertuzumab/trastuzumab	1	1
**Tirosine kinase (TK) inhibitors**		
Lapatinib	1	1
Tucatinib	0	0
**VEGF inhibitors (vascular endothelial growth factor inhibitor)**		
Bevacizumab	1	1
**CDK 4/6 inhibitors (cyclin-dependent kinase 4 and 6)**		
Ribociclib	1	1
Palbociclib	1	1
Abemaciclib	1	1
**PARP inhibitors [poly (ADP-ribose) polymerase]**		
Talazoparib	1	1
Olaparib	1	1
**PD-L1 inhibitors (PD-1 and PDL1 immune checkpoint proteins inhibitors)**		
Atezolizumab	0	1
Pembrolizumab	1	1
**PI3K**α**-specific inhibitors (PI3K catalytic subunit p110**α **inhibitors)**		
Alpelisib	0	1
**Trop-2-directed antibody and topoisomerase inhibitor**		
Sacituzumab govitecan	0	0

**Figure 1:**
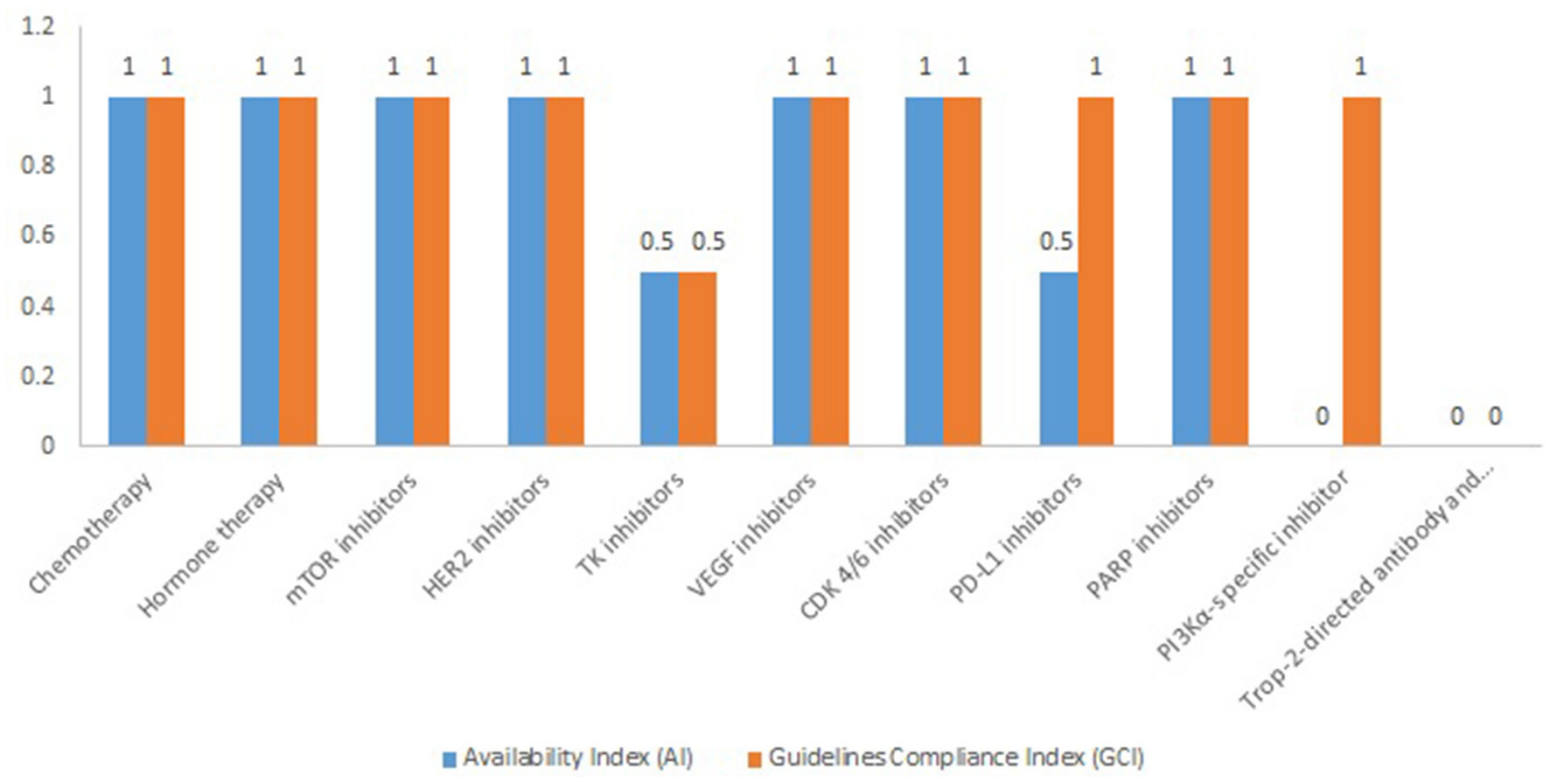
Availability index and compliance index of compared pharmaco-therapeutic groups.

The average value of AI and GCI of chemotherapy medicines is 1, while the average values for targeted therapy are 0.67 for AI and 0.83 for GCI, respectively. The highest AI and GCI values are observed for mTOR inhibitors, HER2 inhibitors, VEGF inhibitors, CDK 4/6 inhibitors, and PARP inhibitors (Figure 1).

Currently from the PDL-1 inhibitors, only pembrolizumab received one positive decision for PDL with an indication of ABC at the end of 2022, but both pembrolizumab and atezolizumab are included in the pharmaco-therapeutic guidelines for triple-negative breast cancer. Alpelisib has only a registered price.

The W.A.I.T. indicator varies from 69 days to 1,381 days. Earlier access is more prominent for biosimilar medicines that entered the Bulgarian pharmaceutical market through the reimbursement system for < 100 days after marketing authorization (Table 3).

**Table 3:** Time to market access for advanced breast cancer targeted therapy in Bulgaria.

**INN**	**EMA (European medicines agency) first approval date**	**EMA (European medicines agency) approval date for ABC**	**ESMO (European society for medical oncology) guidelines**	**PDL (positive drug list) inclusion date for ABC indication**	**W.A.I.T. (waiting to access innovative therapies) (days) for ABC**
**mTOR inhibitors**					
Everolimus	02/08/2009	21/06/2012	√	22/02/2013	246
**HER2 inhibitors**					
Trastuzumab biosimilar^*^	16/05/2018	16/05/2018	√	2/08/2018	78
Trastuzumab deruxtecan	18/01/2021	18/01/2021	√	22/12/2022	703
Trastuzumab emtansine	15/11/2013	15/11/2013	√	11/10/2015	695
Pertuzumab	04/03/2013	04/03/2013	√	25/9/2014	570
Pertuzumab, trastuzumab	21/12/2020	21/12/2020	√	29/12/2021	373
**TK inhibitors**					
Lapatinib	10/06/2008	10/06/2008	√	22/3/2012	1,381
Tucatinib	11/02/2021	11/02/2021	√	NA	
**VEGF inhibitors**					
Bevacizumab biosimilar^*^	26/03/2021	26/03/2021	√	3/6/2021	69
**CDK 4/6 inhibitors**					
Abemaciclib	26/09/2018	26/09/2018	√	25/11/2019	425
Palbociclib	09/11/2016	09/11/2016	√	28/12/2017	414
Ribociclib	22/08/2017	22/08/2017	√	27/9/2019	766
**PD-L1 inhibitors**					
Atezolizumab	20/09/2017	27/06/2019	√	NA	
Pembrolizumab	17/07/2015	16/09/2021	√	22/12/2022	462
**PARP inhibitors**					
Talazoparib	20/06/2019	20/06/2019	√	20/05/2022	1,065
Olaparib	16/12/2014	28/02/2019	√	18/12/2020	659
**PI3K**α**-specific inhibitor**					
Alpelisib	27/07/2020	27/07/2020	√	NA	
**Trop-2-directed antibody and topoisomerase inhibitor**					
Sacituzumab govitecan	22/11/2021	22/11/2021	√	NA	

^*^In the analysis, we included only the biosimilars trastuzumab (Kanjinti) and bevacizumab (Oyavas) with the fastest time to market access.

The average time to market access from marketing authorization to inclusion in PDL for targeted therapy, measured with the W.A.I.T. indicator, is 647 days (SD = 318.07, standard deviation) without considering the biosimilars and 564 days (SD = 359.04 standard deviation) when including the biosimilars in the overall analysis. The shortest time for the therapeutic group is observed for VEGF inhibitors −69 days, but for the observed period in this group, only biosimilars entered the market. For innovative therapies, the shortest average time to access is observed for mTOR inhibitors −246 days.

The analysis of the financial mechanisms for market access in Bulgaria shows that regulatory requirements for pricing and reimbursement are harmonized across therapeutic groups and there are no specific criteria for individual oncology medicines (11, 12). In addition, the cost of the medicinal product is the same for all its indications—there are no differentiated pricing levels depending on the therapeutic indication or the number of patients to be treated. A serious barrier to market access in breast cancer is also the negotiation of rebates with the Fund—the lack of flexibility and variety in the type of agreement indicates that only the financial aspect of treatment is considered when signing them but not the outcomes for each individual patient. A previously conducted analysis on the budget-cap and payback models in Bulgaria shows that the upfront payment requirement and payback approach further distort the ability of marketing authorization holders to forecast budgets and the unstable health system provides no incentive to keep products on the market in the country (21).

## 4. Discussion

To our knowledge, this is the first study at the national level assessing the treatment patterns and mechanisms of patient access to medicines for breast cancer in Bulgaria. A recent systematic review focused on low- and middle-income countries showed wide variations in the prices of cancer medicines, which make them less affordable for patients with low-income levels. The higher cost of cancer medicines, the type of national public insurance schemes, and the non-availability of the facilities are the main established barriers to therapy access (22). Funding of oncology medicines is nationally regulated both for outpatient and hospital treatments (23). The studies reported substantial differences in anticancer medicines included in the Essential Medicines List, availability in private and public hospitals, and the level of reimbursement (24, 25). The cost of anticancer medicines and market approval are among the main factors contributing to inequity between countries.

As a positive feature of the present study, we could consider the application of the AI and the W.A.I.T. indicators that provide the possibility to compare the current state of patient access to oncology medicines with other countries. We provide a more detailed glance at the level of compliance with international guidelines.

Time to market and patient access to advanced breast cancer therapy in Bulgaria are provided within 1–2 years −564 days, on average, varying from 69 days to 1,381 days. These results are in line with those of other already published studies. An analysis performed by the European Federation of Pharmaceutical Industries and Associations (EFPIA) ranks Bulgaria 26th out of a total of 34 European countries, with an average of 611 days to gain access to patients to oncology medicines (15).

An Italian study evaluating the time to patient access of oncology medicines approved by EMA in 2006–2008 shows that the overall required time was 2.3 years. In total, 31.85% of the time to patient access was attributed to the scientific evaluation made by the EMA, followed by local procedures for market access performed by the Agenzia Italiana del Farmaco (AIFA) −28.2%. The time to patient access varied for the different medicines approved in the aforementioned period. Those medicines that were authorized with a risk-sharing agreement, however, had earlier patient access in Italy (26).

In Slovenia, the time to market access of innovative oncology medicines exceeds 2 years. A study shows that, for these medicines, it is almost the same as the time to market access to other medicines and to those with no substantial clinical benefit (27).

To bridge the gap between marketing authorization and patient access, at the regional level, EMA developed some mechanisms for early access programs, especially in the field of oncology (28). The adaptive pathways are such mechanism to set early engagement of drug regulators, HTA bodies, and manufacturers. Adaptive pathways provide the possibility for conditional approval with public coverage with shared risk and early market access, especially in areas with increased medical needs (29).

The results of the current study also show that, in terms of international guidelines, compliance treatment of advanced breast cancer in Bulgaria follows the recommendations of the European Society of Medical Oncology (ESMO) and confirms the results of a previously conducted study assessing the treatment practices of patients with breast cancer in the Central-Eastern European region (30). Currently, four medicines are not available in Bulgaria for advanced breast cancer through the reimbursement systems—sacituzumab govitecan, tucatinib, atezolizumab, and alpelisib. Alpelisib has registered price as of July 2021. However, atezolizumab, sacituzumab govitecan, and tucatinib are included in the pharmaco-therapeutic guideline for metastatic disease and we expect subsequent PDL inclusion in the near future. Trastuzumab deruxtecan and pembrolizumab received one positive decision for PDL inclusion at the end of 2022 and will be paid with public expenditure as of January 2023.

The present study has a few limitations. The first limitation is that, before 2019, oncology medicines were included in the Positive Drug List according to marketing authorization and before this year, we cannot indicate the exact date of inclusion of certain therapeutic indications in the reimbursement list. The second limitation of the present study is that we consider the date of PDL inclusion as the date of market access in Bulgaria. However, there is a legislative rule that an innovative medicine should be included in the Positive Drug List before 30 September of the year to receive public coverage from the next year. For a new therapeutic indication, this rule is not valid but the marketing authorization holder has first to negotiate a financial agreement with the National Health Insurance Fund before the actual reimbursement. These circumstances further delay the actual patient access (time to innovation). This rule is not applicable for generic and biosimilar medicines and that is the reason why we observe faster time to market access to the biosimilars in oncology. Moreover for Bulgaria, there is no mechanism for pre-reimbursement access of authorized medicines through named patient programs or other programs with public expenditure. Another important limitation of the present study is that we do not have officially published data and no official patient registry to analyze the survival rates among the different therapies. This information would be of great importance for decision-making and resource allocation.

There is a program for compassionate use in place regulated by the drug law but it is considered only for medicines which are in the process of obtaining a marketing authorization and there are no other available alternatives for patients with cancer.

Based on other countries' experience, the time to market access to medicines with proved unmet medical needs and clinical benefit could be improved with reimbursement decision based on performance-based risk-sharing agreements or the introduction of named patient programs for patients for whom there is no other therapeutic alternative.

Studies show that, despite the centralized procedure for making marketing authorization valid throughout the European Union for oncology medicines, the time to patient access still varies among the EU Member States mainly due to the local legal requirements for pricing and reimbursement decisions. Despite the introduction of health technology assessment (HTA) as an instrument for transparent reimbursement decisions, there are differences in the HTA methodologies applied across European countries (31).

Numerous initiatives are introduced in different countries to find out mechanisms for the reimbursement and funding of new medicines (32). To overcome the aforementioned barriers and to foster market and patient access, some countries developed methodologies for new market access agreements to reduce the financial burden and cost of uncertainty to public payers by sharing the risk between the payer and the manufacturer (6). Italy and the UK are among the first countries to provide market access agreements for specific medicines, especially in the oncology segment (33).

At the regional level, there are also initiatives for early dialog between pharmaceutical manufacturers, drug regulators, and Health Technology Assessment (HTA) bodies to accelerate patient access to innovative therapies. Joint EMA–HTA scientific advice procedures and the adaptive pathway approach provide possibilities for an early discussion of key aspects of the clinical development of medicines in areas with higher unmet medical needs and also possibilities for financial coverage (34).

## 5. Conclusion

Patient access to advanced breast cancer therapy in Bulgaria follows the most up-to-date recommendations for treatment but the time to access is delayed by 1–2 years since marketing authorization in the European Union. Specific national requirements for pricing and reimbursement and the policy and market access forces of the pharmaceutical companies at the national level are the main factors influencing the time to patient access in Bulgaria. Additional studies should be made to evaluate the time to innovation of the possible role of adaptive pathways and joint HTA–EMA procedures in assuring timely and equal patient access to oncology medicines in the European Union.

## Data availability statement

The raw data supporting the conclusions of this article will be made available by the authors, without undue reservation.

## Author contributions

SK, EP, and BT collected the data and wrote the Results Section. PD, EK, and MD wrote the Introduction and Conclusion. MD wrote the methodology and data validation. ZM and GP contributed to analyzing, writing, and revising. All authors contributed to the article and approved the submitted version.
